# A LiDAR Sensor-Based Spray Boom Height Detection Method and the Corresponding Experimental Validation

**DOI:** 10.3390/s21062107

**Published:** 2021-03-17

**Authors:** Hanjie Dou, Songlin Wang, Changyuan Zhai, Liping Chen, Xiu Wang, Xueguan Zhao

**Affiliations:** 1 Beijing Research Center of Intelligent Equipment for Agriculture, Beijing 100097, China; douhanjie2020@163.com (H.D.); chenlp@nercita.org.cn (L.C.); wangx@nercita.org.cn (X.W.); zhaoxg@nercita.org.cn (X.Z.); 2 National Engineering Research Center of Intelligent Equipment for Agriculture, Beijing 100097, China; 3 College of Mechanical and Electronic Engineering, Northwest A&F University, Yangling 712100, China; 4 College of Mechanical Engineering and Automation, Liaoning University of Technology, Jinzhou 121001, China; wangslxss@163.com

**Keywords:** boom sprayer, boom height control, wheat stubble, wheat stubble root, K-means clustering

## Abstract

Sprayer boom height (*H_b_*) variations affect the deposition and distribution of droplets. An *H_b_* control system is used to adjust *H_b_* to maintain an optimum distance between the boom and the crop canopy, and an *H_b_* detection sensor is a key component of the *H_b_* control system. This study presents a new, low-cost light detection and ranging (LiDAR) sensor for *H_b_* detection developed based on the principle of single-point ranging. To examine the detection performance of the LiDAR sensor, a step height detection experiment, a field ground detection experiment, and a wheat stubble (WS) height detection experiment as well as a comparison with an ultrasonic sensor were performed. The results showed that the LiDAR sensor could be used to detect *H_b_*. When used to detect the WS height (*H_WS_*), the LiDAR sensor primarily detected the WS roots and the inside of the WS canopy. *H_WS_* and movement speed of the LiDAR sensor (*V_LiDAR_*) has a greater impact on the detection performance of the LiDAR sensor for the WS canopy than that for the WS roots. The detection error of the LiDAR sensor for the WS roots is less than 5.00%, and the detection error of the LiDAR sensor for the WS canopy is greater than 8.00%. The detection value from the LiDAR sensor to the WS root multiplied by 1.05 can be used as a reference basis for adjusting *H_b_*, and after the WS canopy height is added to the basis, the value can be used as an index for adjusting *H_b_* in WS field spraying. The results of this study will promote research on the boom height detection method and autonomous *H_b_* control system.

## 1. Introduction

In recent years, as the level of agricultural mechanization in China has increased, boom sprayers have become the primary implements for spraying operations in fields due to their relatively good nozzle atomization performance, relatively large operating widths, and relatively high operating efficiency [1,2,3]. Since a boom sprayer sprays over a relatively wide area and field surfaces are uneven [4], unwanted boom motions typically occur when the sprayer tires pass over uneven terrain in the field, which can change the spraying height, cause the sprayer to fail to meet the operating requirements [5], and result in problems, such as the uneven deposition and distribution of droplets [6,7]. Thus, to improve the uniformity of the application of pesticides and reduce droplet drift during the field spraying process, it is necessary to maintain an optimum distance between the boom and the crop canopy [8,9,10]. It is vitally important to monitor and adjust the distance between the boom and the crop canopy in real-time.

The boom moves primarily by swinging and rotating the boom sprayers. These are the main movement forms that affect the deposition and distribution of droplets in fields. Researchers in China and abroad have conducted a series of studies on the effects of boom movement on the deposition and distribution of droplets and found that a significant difference between the two ends of spray boom can lead to a high coefficient of variation for the distribution of droplets. The number of droplets deposited by a sprayer with a boom swing of 0.3 m can reach three times that deposited by a normal spraying process [11,12]. Langenakens et al. studied the influence of the boom height vibration on droplet deposition and distribution through simulation methods. The results showed that the horizontal movement of the boom caused the variation coefficient of the droplet distribution to change between 20% and 600%, and the vertical vibration of the boom caused the variation coefficient of the droplet distribution to change between 0% and 1000% [13]. The boom height (*H_b_*) can be detected in real-time with sensors, Table 1 lists the sensors currently used to detect the boom motion parameters. The ultrasonic sensors are the primary sensors used to detect *H_b_*, and the acceleration sensors are used to measure boom vibration. Based on the sensors in Table 1, some sensor systems are developed to effectively detect *H_b_*, which makes it possible to adjust the boom of a sprayer to follow the ground contour, thus maintaining the distance between the boom and ground or distance between the boom and the crop canopy within a stable range as the sprayer passes through an area with a complex and varied terrain. Each John Deere sprayer uses three ultrasonic sensors and one tilt sensor to achieve automatic detection of the position and attitude of the boom. Each Case sprayer uses ultrasonic sensors and adjusting wheels to control *H_b_*. Each Amazone sprayer achieves autonomous control and intelligent adjustment of the height and posture of the boom through tilt and ultrasonic sensors.

Ultrasonic sensor-based autonomous *H_b_* control systems have been developed. Cui et al. [14,15] developed an automatic *H_b_* control system based on a digital signal processor (DSP) and proposed a signal processing method that integrates limit filtering, data smoothing, and an optimum weight-based data fusion algorithm. This system can effectively control changes in *H_b_* and meet field operating requirements. Magalhães et al. [23] controlled changes in *H_b_* using a fractional proportional-integral-derivative (PID) control technique that was more suitable for *H_b_* control than conventional PID control techniques. Tahmasebi et al. [24,25] controlled the boom movement using a robust intelligent control method based on active torque control (ATC). This method can effectively impede the swing and vibration of a boom relative to its suspension system. However, these autonomous *H_b_* control systems perform relatively well on flat terrain and plains with large areas of farmland but are unsuitable for high-speed, complex operating settings. Complex farmland grounds require that autonomous *H_b_* control systems have relatively high response speeds; however, ultrasonic sensors are prone to disturbance from external factors during the *H_b_* detection process, and there are bottlenecks in the research on signal processing algorithms for ultrasonic sensors for high-speed or complex operating settings. As boom sprayers are becoming increasingly large and efficient, it is urgently necessary to make breakthroughs in signal processing algorithms for *H_b_* detection sensors or to develop new sensors suitable for *H_b_* detection.

The objective of this research was to develop a new, low-cost light detection and ranging (LiDAR) sensor for *H_b_* detection based on the principle of single-point ranging. A step height detection experiment, a field ground detection experiment, and a wheat stubble (WS) height detection experiment as well as a comparison with an ultrasonic sensor were performed to evaluate the detection performance of the LiDAR sensor. Meanwhile, a *H_b_* detection method based on the LiDAR sensor was proposed to adjust the boom height for spraying on WS fields. The results of this study support research on boom height detection method and autonomous *H_b_* control system.

This paper is structured in five sections, as follows: Section 1 introduces the previous work and the purpose of the research. Section 2 provides the LiDAR sensor design, experimental platform design for *H_b_* detection and the detection performance experiment for the LiDAR sensor. Section 3 presents the field test results. Section 4 discusses the test results. Section 5 presents the conclusions.

## 2. Materials and Methods

### 2.1. LiDAR Sensor Design

**Figure 1:**
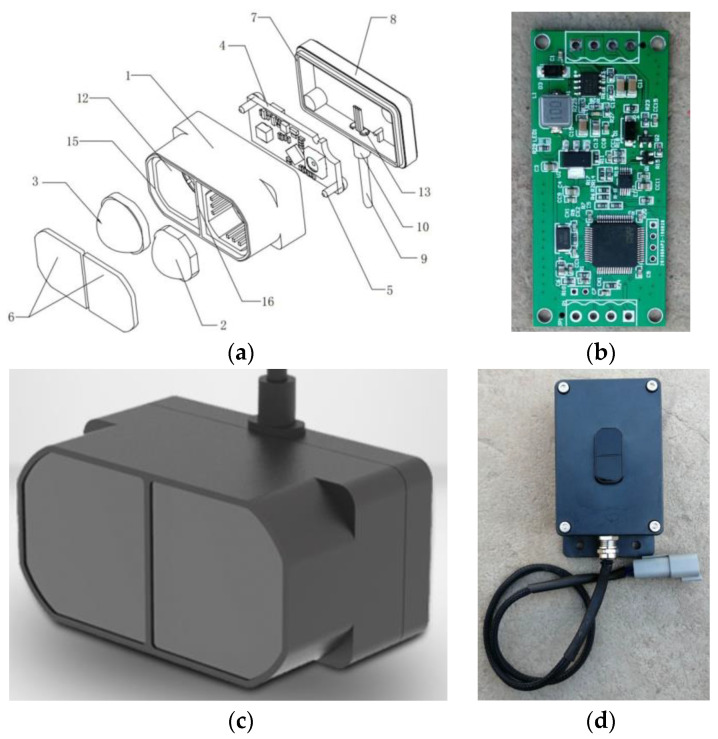
LiDAR sensor: (**a**) Structural schematic of the sensor, 1. Front shell; 2. Transmitting lens; 3. Receiver lens; 4. Circuit board; 5. Bolt; 6. Lens; 7. Ultrasonic welded structure; 8. Back shell; 9. Tail cable; 10. Tail cable fastener; 11. Location column; 12. Mounting hole; 13. Barbed structure; 14. Protective shield; 15; Mounting grooves; 16. Partition structure. (**b**) Signal processing circuit board. (**c**) LiDAR sensor sealed enclosure. (**d**) Final encapsulated sensor.

Compared to ultrasonic sensors, LiDAR sensors have advantages, such as a higher measurement precision, a higher response speed, and a higher interference resistance. In this study, a new LiDAR sensor suitable for *H_b_* detection was developed based on a low-cost, small-sized, low-power single-point laser rangefinder, TFmini Plus, manufactured by Benewake Co., Ltd. (Beijing, China), which measures distances based on the time-of-flight (ToF) principle. During the measurement process, the sensor periodically outwardly transmits a modulated near-infrared wave that is reflected upon contact with an object. The ToF is calculated by determining the difference in phase between the incident and reflected waves. On this basis, the distance from the tested target is determined. This single-point ranging sensor is composed primarily of a front shell, a transmitting lens, a receiver lens, a circuit board, screws, lenses, an ultrasonic welded structure, a back shell, a tail cable, a tail cable fastener, location columns, a mounting hole, a barbed structure, a protective shield, mounting grooves, and a partition structure (Figure 1a). Equipped with a unique optical and electrical design, this sensor can measure distances in a stable, accurate, and highly sensitive manner. The parameter indicators of the LiDAR sensor are shown in Table 2.

The single-point LiDAR sensor outputs signals at the transistor-transistor logic (TTL) level and thus has a limited signal transmission distance. *H_b_* detection sensors installed at the two ends of a boom transmit signals over a distance exceeding 10 m, but the TTL transmission mode cannot ensure signal validity. To address this problem, a signal processing module was developed based on an STM32F103RCT6 single-chip microcomputer (SCM). This module can convert the signal output of the LiDAR sensor to a 4–20 mA signal output. Figure 1b shows the circuit board of this module. This signal processing module primarily consists of a main control module, a power module, and a current module. The main control module, composed of an ST STM32F103RCT6 SCM with a 72-MHz central processing unit (CPU) and 1-MB flash memory, is capable of simultaneously supporting serial port and controller area network (CAN) communication. The program download module has a serial wire debug (SWD) download interface that allows the user to develop and debug programs. The power module uses MPS MP1584DN and AMS AMS1117-3.3 switch mode voltage regulators and can supply a 12 V direct current (DC) and output 5 V and 3.3 V. The current output module, composed of a Texas Instruments voltage-to-current converter chip, is capable of converting a sensor signal to a 4–20 mA current signal output. During operation, the power module converts a 12 V DC input voltage to 5 V and 3.3 V to supply power to the main control and current output modules. The main control module acquires the distance information measured by the LiDAR sensor through communication at the TTL level via the serial port and subsequently converts it to a voltage signal and outputs it to the current output module via a pin of the SCM. The current output module converts the voltage signal to a current signal output. The detection range of the LiDAR sensor of 0.1–12 m corresponds to a current signal range of 4–20 mA. To improve the stability and accuracy of the output current signal in the signal conversion process, a PID control algorithm was incorporated into the signal conversion algorithm. In addition, to enhance the ranging performance of the LiDAR sensor in complex operating conditions, an adaptive algorithm for multiple application settings and targets was added to the signal conversion process, and multiple adjustable configurations and parameters were included. These measures can ensure that the LiDAR sensor performs well in ranging operations in complex settings and meets agricultural requirements in complex applications. Finally, the LiDAR sensor was encapsulated to facilitate the mounting and protection, as shown in Figure 1c,d. The boom sprayers often operate in complex field conditions. The encapsulated sensor was subjected to environmental temperature testing at −40 to 85 °C, vibration testing, and Ingress Protection 67 testing to determine if it could meet the operating requirements.

### 2.2. Sensor Check

**Figure 2:**
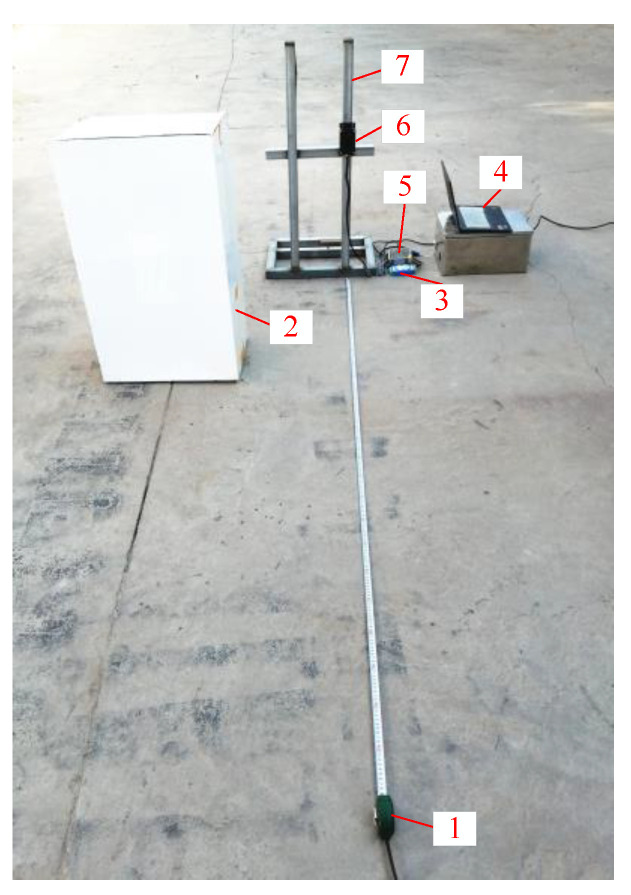
LiDAR sensor check experiment: 1. Tape measure; 2. Paper box 3. Current signal acquisition card; 4. Laptop computer; 5. Power source; 6. LiDAR sensor 7. Fixation frame.

The LiDAR sensor needs to be checked prior to use. Figure 2 shows a photograph of the experimental setting for checking the LiDAR sensor. The LiDAR sensor was placed in an unobstructed, open space. A computer was connected via a universal serial bus (USB) serial port to a current signal acquisition card. The computer interface could display the current signal output by the LiDAR sensor in real time. A paper box was used as an object for detection in the experiment. A tape measure was placed directly below the central axis of the beam transmitted by the LiDAR sensor to record the distance over which the paper box moved. During the experiment, the paper box was placed directly in front of the sensor. The paper box was moved at distance intervals of 10 cm within the measuring range (0.1–2 m) of the LiDAR sensor. The current signal output by the LiDAR sensor was recorded when the paper box was moved. Three repeats were performed for each process. The average of the three measurements was used to fit a detected distance-output current curve.

### 2.3. Experimental Platform Design for H_b_ Detection

**Figure 3:**
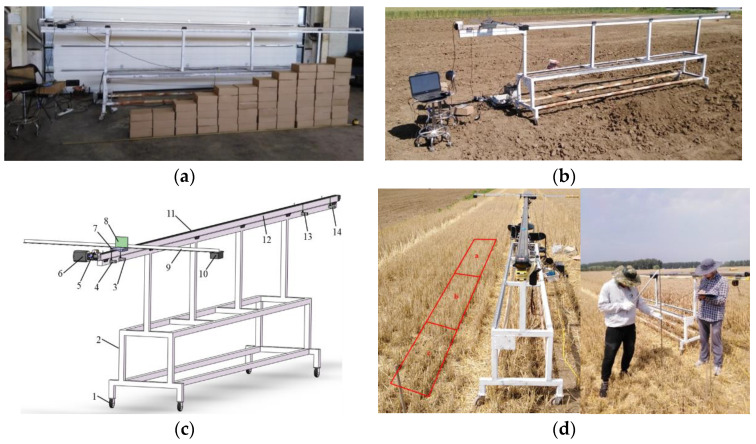
Detection performance experiment of the LiDAR sensor. (**a**) Setup for the step detection experiment. (**b**) Setup for the field ground detection experiment. (**c**) Structural diagram of the experimental platform for *H_b_* detection, 1. Mecanum wheel; 2. Support frame; 3. Limit switch 1; 4. Limit switch 2; 5. Laser rangefinder; 6. Stepper motor; 7. Sliding block; 8. Laser-reflecting plate; 9. Sensor fixation bar; 10. LiDAR sensor; 11. Synchronous belt; 12. Sliding track; 13. Limit switch 3; 14. Limit switch 4. (**d**) Setup for the height detection experiment in a WS field.

The detection performance of the LiDAR sensor was mainly affected by the moving speed and the uneven wheat stubble canopy. To investigate the effect of moving speed and uneven wheat stubble canopy on the detection performance of the LiDAR sensor, an experimental platform for *H_b_* detection by the LiDAR sensor was constructed, as shown in Figure 3c. This experimental platform was composed primarily of a sensor fixation bar, a sliding track, a stepper motor, a synchronous belt, a sliding block, a stepper motor driver, and a controller. The total length of the experimental platform was 6.5 m. The effective length of the sliding track was 6.0 m. The sliding track was fixed in a bridge mode. The sliding block, with a length of 0.3 m, was used to fix the sensor fixation bar. Four CZ-7166 limit switches (Testo Instruments International Trading (Shanghai, China) Co., Ltd.), including two outer limit switches and two inner limit switches, were set at the two ends of the sliding track. The two outer limit switches, which served as safety limit switches, prevented the sliding block from impacting the two ends of the sliding track. The two inner limit switches, which served as position limit switches, limited the range of movement of the sliding block to 5 m. Of the mobile devices, the KH-01 stepper motor controller (Relong Automation Equipment (Zhejiang, China) Co., Ltd.) precisely controlled the HB80D stepper motor (Hongbaoda Industry (Shenzhen, China) Co., Ltd.) via the HB860D driver (Hongbaoda Industry Co., Ltd.) to drive the sliding block to steadily move along the track. The speed and range of the sliding block could be precisely controlled. During the experiment, to accurately record *H_b_* values detected by the LiDAR sensor at different locations in the horizontal direction, an S85-MH-5-Y03-OOI laser rangefinder with a measuring range of 10 m and a current signal output of 4–20 mA (Datalogic Industrial Automation Company Ltd. (Shenzhen, China)) was fixed at one end of the sliding platform. The laser-reflecting plate and the LiDAR sensor were placed at the same horizontal location. The laser rangefinder recorded the horizontal location of the LiDAR sensor by measuring the location of the laser-reflecting plate. An SG-AD-Modbus-8I data acquisition card (DAC) (Sange Electronic Technology (Tianjin, China) Co., Ltd.) was used to simultaneously collect measurement data from the laser rangefinder and the LiDAR sensor. The DAC communicated with the upper computer via a RS485 serial port. A general acquisition system for analogue signals designed by Zhai et al. [26] that can collect experimental data and store the data in a database to facilitate subsequent experimental analysis was used in the upper computer.

### 2.4. Step Detection Experiment

To examine the effects of the movement speed of the LiDAR sensor (*V_LiDAR_*) on the detection performance, a step detection experiment was designed. In this experiment, 70 paper boxes (0.30 m × 0.20 m × 0.10 m) were stacked to form a flight of eight steps, as shown in Figure 3a. The LiDAR sensor was fixed at one end of the sensor fixation bar and could detect vertically downward (Figure 3a). Prior to the experiment, the sliding block of the experimental platform for *H_b_* detection was manually pushed to change the distance detected by the LiDAR sensor. A tape measure was used to measure the distance of the LiDAR sensor from the ground and each step, and the corresponding measurements were recorded. During the experiment, the center of the step-like ground for detection was placed on a straight line parallel to and at a horizontal distance of 1.0 m from the center line of the track of the sliding platform. The sliding block was used to drive the LiDAR sensor to move along a straight line at a uniform speed. The LiDAR sensor was located directly above the surface of the steps for detection at the site throughout its movement with the sliding block. In addition, the position limit switches were used to control the LiDAR sensor in such a way that the LiDAR sensor took measurements at distance intervals of 5.0 m. According to the experimental design in Zhai et al. [26], the measurements taken by the LiDAR sensor when moving at a speed of 0.05 m/s were treated as true values. The sliding block was controlled to move at speeds of 0.3, 0.6, 0.9, and 1.2 m/s. The measurements taken by the LiDAR sensor when moving at various speeds were collected. Three repeats were performed for each test. The average of the three measurements taken by the LiDAR sensor was used as the ultimate measured value.

### 2.5. Field Ground Detection Experiment

A field ground detection experiment was performed in a rotary-tilled field, as shown in Figure 3b. In this experiment, the distance between the signal transmission plane of the LiDAR sensor and the ground was measured. The experimental platform for *H_b_* detection was placed in a rotary-tilled field. The distance between the LiDAR sensor and the ground was set to 1.4 m. Soil was manually heaped to produce a small slope, approximately 0.5 and 0.4 m in length and height, respectively, on the ground that was a horizontal distance of 1.0 m from the centre line of the track of the sliding platform to simulate an undulating surface. The sliding block was used to drive the LiDAR sensor to move over 5.0 m along a straight line at a uniform speed directly above the horizontal line of the small slope, as shown in Figure 3b. During the experiment, a tape measure was first used to measure the distance between the LiDAR sensor and the ground at intervals of 0.1 m. Then, *V_LiDAR_* was adjusted to 0.05 m/s. The measurements taken by the LiDAR sensor when moving at this speed were treated as the true values. Finally, the sliding block was controlled to move at speeds of 0.3, 0.6, 0.9, and 1.2 m/s. The measurements taken by the LiDAR sensor at various speeds were collected. Three repeats were performed for each test. The average of the three measurements taken by the LiDAR sensor was used as the ultimate measured value.

### 2.6. Height Detection Experiment in a WS Field

The autumn crops are generally planted without tillage after harvesting wheat in China. It is necessary to spray pesticides and remove weeds before no-tillage planting. To examine the effects of WS canopies on the *H_b_* detection performance of the LiDAR sensor, an *H_b_* detection experiment in a post-harvest WS field was designed, as shown in Figure 3d. Before the experiment, an unharvested wheat plot 5.0 m long and 0.3 m wide was randomly selected. A harvester was then used to harvest the wheat in the plot. The header height of the harvester was set to 0.35, 0.25, and 0.15 m for areas a, b, and c of the plot, respectively, as shown in Figure 3d. Post-harvest areas a, b, and c were 1.5, 2.2, and 1.3 m in length, respectively. During the experiment, the experimental platform for *H_b_* detection was first placed in each WS area in such a way that the LiDAR sensor was directly above the selected WS canopy. After the experimental platform was placed, the LiDAR sensor was moved to the starting position. A tape measure was used to measure the distances of the LiDAR sensor from the WS canopy and the ground at intervals of 0.1 m until the LiDAR sensor moved to its final position. Subsequently, *V_LiDAR_* was adjusted to 0.05 m/s, and the measurements taken by the LiDAR sensor when moving at this speed were treated as the true values. Finally, the sliding block was controlled to move at speeds of 0.3, 0.6, 0.9, and 1.2 m/s. The measurements taken by the LiDAR sensor at these speeds were collected. Three repeats were performed for each test. The average of the three measurements taken by the LiDAR sensor was used as its ultimate measurement.

### 2.7. Comparison of the LiDAR Sensor and an Ultrasonic Sensor

**Figure 4:**
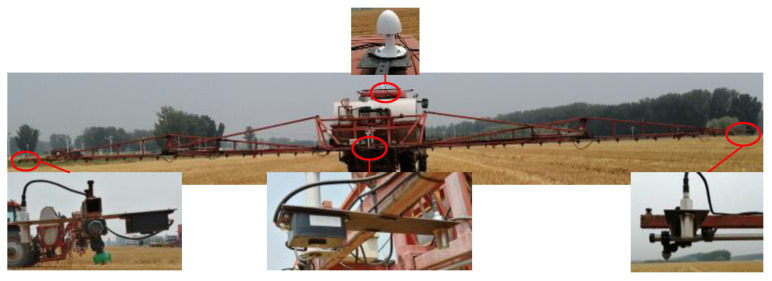
Knapsack boom sprayer.

The ultrasonic sensors are the main type of sensor used to detect *H_b_*. To better illustrate the feasibility of using the LiDAR sensor to detect *H_b_*, an experiment was designed to compare the LiDAR sensor with an ultrasonic sensor. A knapsack boom sprayer was selected for the experiment. The boom of this sprayer was 21 m in length and was divided into three sections, namely, left, middle, and right sections. The left and right sections of the boom were each further divided into two sections. The extension and contraction of the boom was controlled by a hydraulic cylinder (Figure 4). One LiDAR sensor and one ultrasonic sensor were placed at the left, middle, and right sections of the boom to monitor the changes in *H_b_* in each section in real-time.

**Figure 5:**
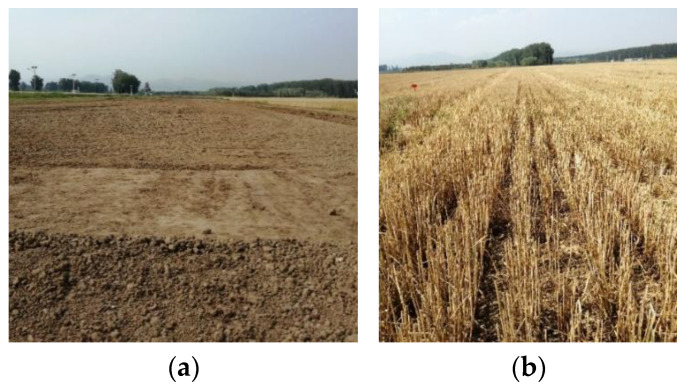
Experimental fields. (**a**) Rotary-tilled field. (**b**) WS field.

The experiment was performed in a rotary-tilled field (400 m × 50 m) and a WS field (560 m × 240 m) with a WS height (*H_WS_*) of 20 cm at the National Research Station for Precision Agriculture, Beijing, China, as shown in Figure 5. During the experiment, the tank of the sprayer was filled half full with water. The height of the nozzle of the sprayer head was set to 110 cm above ground. The signal transmission plane of the LiDAR sensor and the nozzle were set on the same horizontal plane. The sprayer was mounted on a tractor. The Chinese standard GB/T 20183.3-2006 “Equipment for crop protection—Spraying equipment—Part 3: Test methods for volume/hectare adjustment systems of agricultural hydraulic pressure sprayers” is considered, which requires that the speed during the test be 1.5 m/s to 2.5 m/s, and studies have usually sprayed at a moving speed of 1.9 m/s in recent years [27]. The sprayer was operated at speeds of 2.0 m/s. The experiment was repeated three times. The changes in *H_b_* during each test were recorded.

### 2.8. Acceleration Data Processing

The K-means clustering algorithm was used to process the LiDAR sensor detection data. The algorithm first selects k objects at random from data objects as the initial clustering centres, calculates the distance between each object and these central objects according to the mean value of each clustering object, and re-divides the corresponding objects according to the minimum distance. Then, the algorithm re-calculates the average of each cluster. This process is repeated until each cluster no longer changes [28,29]. Usually, the criteria for the K-means algorithm use the squared error criterion function, which is defined as:

(1) $$E=\overset{k}{\underset{i=1}{\sum }}\underset{x\in C_{i}}{\sum }{\left(x-\bar{x_{i}}\right)}^{2}$$

where *E* is the sum of the squared error in the data set of all the LiDAR sensor detection values, $C_{i}$ is the cluster of LiDAR sensor detection values, x is the LiDAR sensor detection value, and $x_{i}$ is the average value of cluster $C_{i}$. k is the number of clusters.

**Figure 6:**
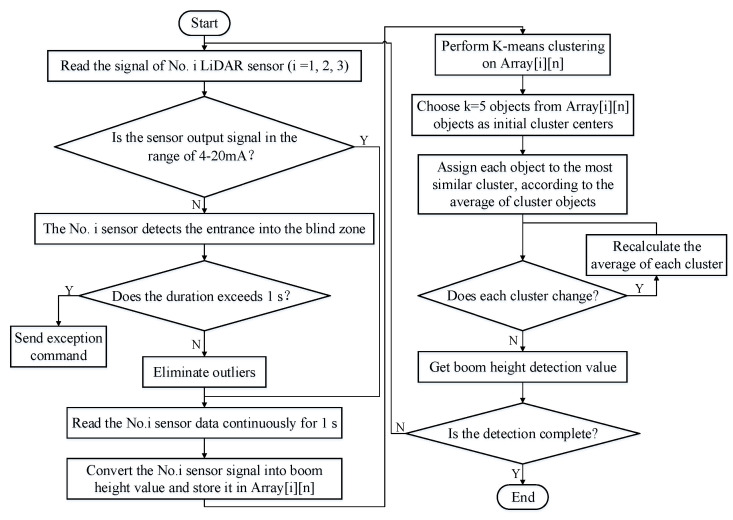
Flow chart of *H_b_* calculation based on the K-means clustering algorithm.

Our previous study contains more details on this process [30]. The flow chart of the *H_b_* calculation based on the K-means clustering algorithm is shown in Figure 6. During the working process, the *H_b_* detection system reads the three LiDAR sensor signals in real-time, and it first judges the validity of the sensor detection signal. If the detection signal is abnormal, it will judge whether the sensor has entered the blind zone. Once the LiDAR sensor has entered the blind zone, the system will send a data exception command. Otherwise, the system will convert the obtained data into *H_b_* values every 1 s and store them in an array form and then call the K-means clustering algorithm to preprocess the acquired *H_b_* detection data, ultimately obtaining accurate *H_b_* detection values.

## 3. Results

### 3.1. Sensor Check

**Figure 7:**
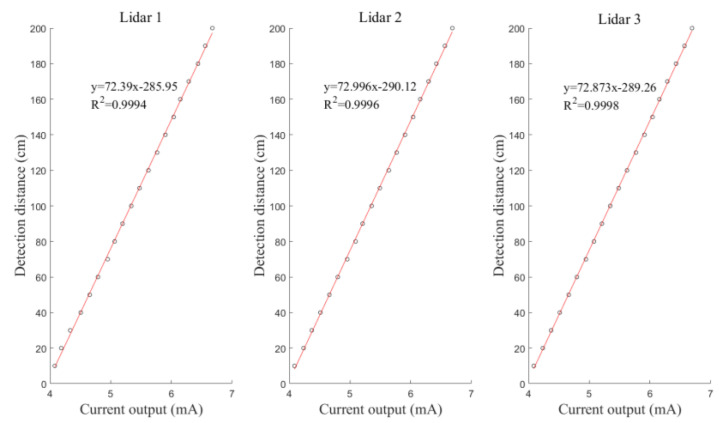
Calibration curves of the LiDAR sensors: where *x* is the output current, mA, and *y* is the actual distance, cm.

The *H_b_* values measured by the LiDAR sensor for the left, middle, and right sections of the boom were fitted to the output current data. Figure 7 shows the fitted curves for the left, middle, and right sections of the boom. The *R^2^* values of the equations are 0.9994, 0.9996, and 0.9998, respectively. Evidently, the three LiDAR sensors show good linearity and meet the operating requirements.

### 3.2. Step Detection Experiment

**Figure 8:**
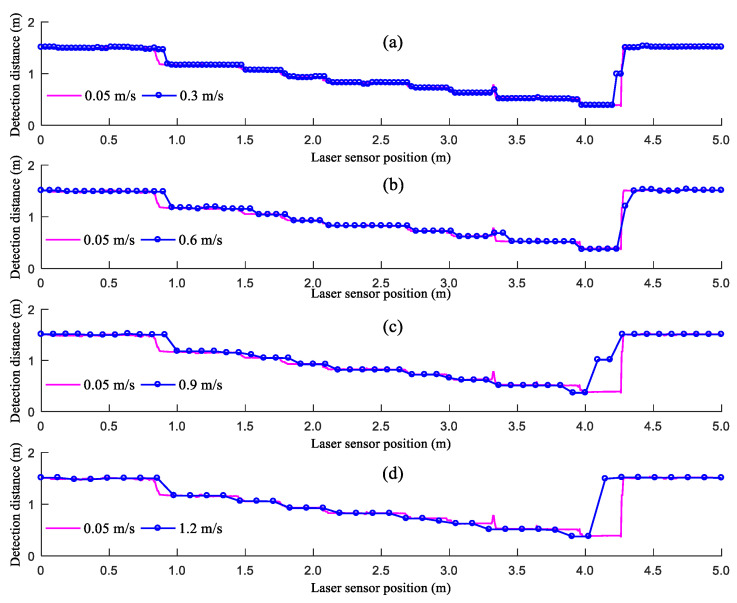
Results of the step detection experiment across a speed gradient: (**a**) 0.3 m/s; (**b**) 0.6 m/s; (**c**) 0.9 m/s; (**d**) 1.2 m/s.

Figure 8 shows the distances detected by the LiDAR sensor when moving at different speeds. As demonstrated in Figure 8, the distances detected by the LiDAR sensor when moving at different speeds can reflect the changes in the step height. However, the distances detected by the LiDAR sensor at the junctions of the ground and the upper surface of the flight of steps formed by paper boxes differ from the true values. In addition, this difference becomes increasingly prominent as *V_LiDAR_* increases. This is because the angle of spread of the beam transmitted by the LiDAR sensor is 3.6°. As the LiDAR sensor moves to directly above each junction of the ground and the upper surface of the flight of steps, the beam transmitted by the LiDAR sensor partially reaches the ground and partially reaches the upper surface of the flight of steps, thus affecting the distance detected by the LiDAR sensor. As the detection of the LiDAR sensor moves from the ground to the upper surface of the flight of steps, there is a detection delay in the LiDAR sensor. Moreover, as *V_LiDAR_* increases from 0.3 to 0.9 m/s, the detection delay increases. As *V_LiDAR_* increases from 0.9 to 1.2 m/s, the detection delay decreases. This is because as *V_LiDAR_* increases, there is a decrease in the amount of data sampled over the same distance of movement, and the low amount of sampled data increases the detection delay. The junction between the ground and the upper surface of the flight of steps is relatively small. When moving at a speed of 1.2 m/s, the LiDAR sensor is unable to detect the junction; therefore, the detection delay is reduced. As the LiDAR sensor moves from the upper surface of the flight of steps to the ground, the distances detected by the LiDAR sensor are between those from the ground and the upper surface of the flight of steps. This is because the large difference between the heights of the ground and the upper surface of the flight of steps at this junction increases the probability for the beam transmitted by the LiDAR sensor to reach the sides of the flight of steps. A low *V_LiDAR_* leads to a high probability that the beam transmitted by the LiDAR sensor reaches the sides of the flight of steps.

### 3.3. Field Ground Detection Experiment

**Figure 9:**
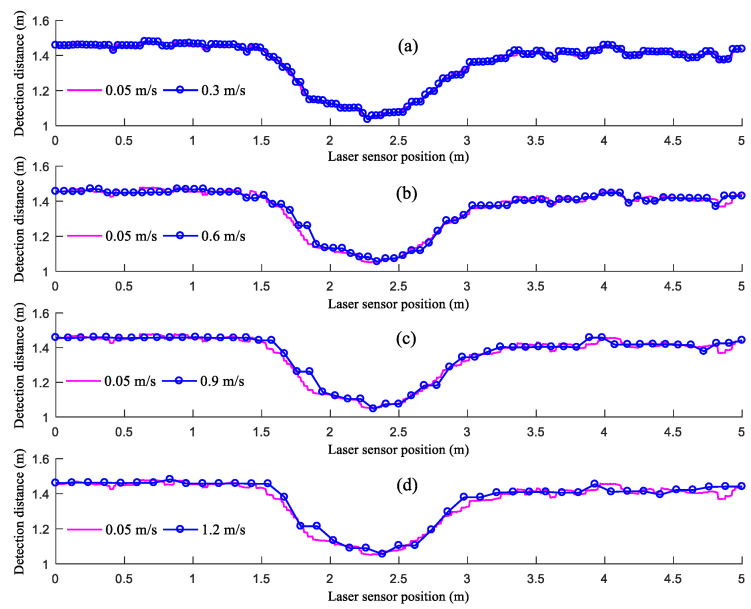
Results of the field ground detection experiment across a speed gradient where: (**a**) 0.3 m/s; (**b**) 0.6 m/s; (**c**) 0.9 m/s; (**d**) 1.2 m/s.

Figure 9 shows the distances from the field ground detected by the LiDAR sensor at different detection speeds. As demonstrated in Figure 9, the distances detected by the LiDAR sensor can reflect the changes in the slope of the field ground well. Thus, the LiDAR sensor can be used to detect changes in *H_b_* in rotary-tilled fields. Compared to the flight of steps used in the step detection experiment, the height of the surface of the slope in the field varies gently. There is no notable detection delay as *V_LiDAR_* increases. However, the distances detected by the LiDAR sensor at the slope differ from the true values. In addition, this difference increases as *V_LiDAR_* increases. This is because as *V_LiDAR_* increases, there is a decrease in the amount of sampled data. The low amount of sampled data reduces the detection accuracy of the LiDAR sensor and, thereby, increases its detection error.

### 3.4. WS Height Detection Experiment

**Figure 10:**
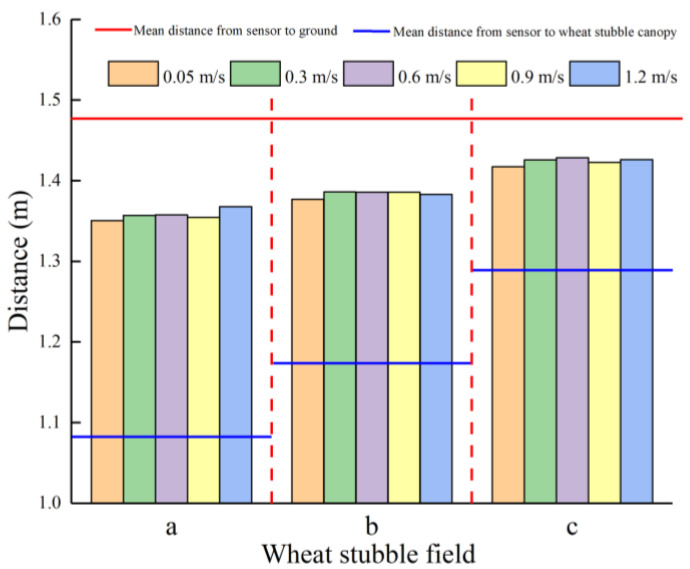
Distances detected by the LiDAR sensor at different *H_WS_* and *V_LiDAR__._*

Figure 10 shows the distances detected by the LiDAR sensor at different *H_WS_* and *V_LiDAR_* values. As demonstrated in Figure 10, when *H_WS_* remains unchanged, there is a small change in the distance detected by the LiDAR sensor as *V_LiDAR_* increases from 0.05 to 1.2 m/s. This suggests that when *H_WS_* remains unchanged, *V_LiDAR_* has less of an impact on the distance detected by the LiDAR sensor. As *H_WS_* increases, the distance detected by the LiDAR sensor increases. The detected distances can reflect the changes in *H_WS_*. However, the distance detected by the LiDAR sensor is between the ground and the surface of the WS canopy. In addition, there is no notable pattern between the changes in the distance detected by the LiDAR sensor and *H_WS_*. This finding suggests that *H_WS_* affects the distance detected by the LiDAR sensor and that the distance detected by the LiDAR sensor is between the ground and the surface of the WS canopy.

**Figure 11:**
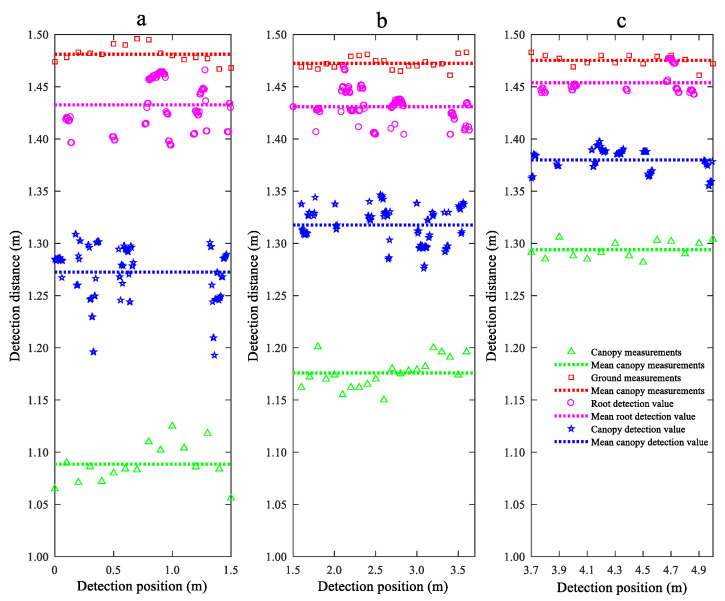
Detection results of LiDAR sensor for different WS heights.

To further analyse the distribution of the distance detected by the LiDAR sensor within the WS canopy, the distances detected by the LiDAR sensor at different *H_WS_* values were subjected to a cluster analysis using the K-means clustering analysis algorithm, and the results are shown in Figure 11. The clustering results show that the detection signal transmitted by the LiDAR sensor partially reaches the inside of the WS canopy and partially reaches the WS roots and that the signal reflection sites are distributed in a random manner. Since the post-harvest wheat straws are thin-walled circular tubes, the WS canopy exhibits a sparse, porous structure that absorbs the detection beam and, thereby, causes the reflection sites of the signal transmitted by the laser within the WS canopy to change. Moreover, due to tillering, there are many branches at the WS roots near the ground, resulting in a relatively high density at the WS roots. Most of the signals transmitted by the LiDAR sensor reach the WS roots. Consequently, the distances from the WS detected by the LiDAR sensor are close to those from the ground.

**Figure 12:**
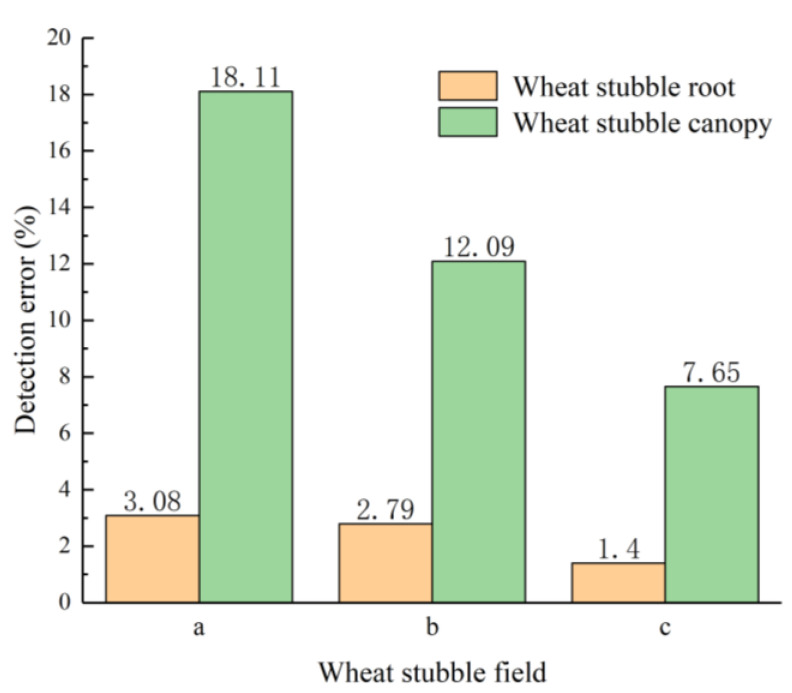
Mean errors of the LiDAR sensor for the WS roots and canopy in each of areas a, b, and c.

To analyse the effects of *H_WS_* on the detection performance of the LiDAR sensor for the WS roots and canopy, the mean errors of the distances detected by the LiDAR sensor from the WS roots and canopy in areas a, b, and c were statistically analysed, and the results are shown in Figure 12. As demonstrated in Figure 12, for each area, the mean error of the distances detected by the LiDAR sensor from the WS roots is lower than that of the distances detected by the LiDAR sensor from the WS canopy. As *H_WS_* decreases, the mean detection errors of the LiDAR sensor for both the WS roots and canopy in each area gradually decrease. The maximum and minimum detection errors of the LiDAR sensor for the WS roots are 3.08% and 1.40%, respectively. There are no significant changes in the detection error of the LiDAR sensor between areas a and b. However, the detection error of the LiDAR sensor for area c is notably lower than those for areas a and b. This is because in areas a and b, the WS canopy is relatively high, and the litter layer at the WS roots had been relatively insignificantly damaged during the harvesting process. In contrast, in area c, the litter layer had been relatively significantly damaged during the harvesting process, so the beam transmitted by the LiDAR sensor mostly reaches the WS roots. As a consequence, the detection error of the LiDAR sensor for the WS roots is demonstrably lower in area c than areas a and b. For the WS canopies, the maximum and minimum detection errors of the LiDAR sensor are 18.11% and 7.65%, respectively, and the detection error of the LiDAR sensor changes considerably in areas a, b, and c. This is because as *H_WS_* decreases, there is an increase in the density of the WS canopy. In addition, the presence of a relatively large amount of litter below a relatively low WS canopy improves the ability of the WS canopy to reflect the laser beam and, thereby, increases the detection accuracy of the LiDAR sensor for the WS canopy.

To investigate the effects of *V_LiDAR_* on the detection performance of the LiDAR sensor for the WS roots and canopy in each area, the detection errors of the LiDAR sensor moving at different speeds for the WS roots and canopy in each area were analysed. Table 3 summarizes the results. As demonstrated in Table 3, *V_LiDAR_* has a greater impact on the detection performance of the LiDAR sensor for the WS canopy than that for the WS roots in each area. For the WS roots in areas a, b, and c, the maximum detection errors of the LiDAR sensors are 4.66%, 3.67%, and 2.44%, respectively, and the minimum detection errors are 2.43%, 2.45%, and 0.68%, respectively. For the WS canopies in areas a, b, and c, the maximum detection errors of the LiDAR sensors are 20.02%, 12.84%, and 8.50%, respectively, and the minimum detection errors are 16.80%, 11.48%, and 6.65%, respectively. When *H_WS_* remains unchanged, *V_LiDAR_* has a slight impact on the detection errors of the LiDAR sensor for the WS roots and canopy. In addition, when *V_LiDAR_* remains unchanged, as the WS canopy height decreases, the detection errors of the LiDAR sensor for the WS roots and canopy decrease.

In summary, *V_LiDAR_* has a small impact on the detection performance of the LiDAR sensor for the WS roots and canopy. However, *V_LiDAR_* has a greater impact on the detection performance of the LiDAR sensor for the WS canopy than that for the WS roots. *H_WS_* has a greater impact on the detection performance of the LiDAR sensor for the WS roots and canopy. Similarly, *H_WS_* has a greater impact on the detection performance of the LiDAR sensor for the WS canopy than that for the WS roots. *H_WS_* and *V_LiDAR_* affect the detection error of the LiDAR sensor for the WS roots but to a relatively small extent. The detection error of the LiDAR sensor for the WS roots is less than 5.00% in all the cases (the maximum detection error is 4.66%). and the detection error of the LiDAR sensor for the WS roots is greater than 8.00% (the minimum detection error is 8.50%).

### 3.5. Comparison of the LiDAR and Ultrasonic Sensors

**Figure 13:**
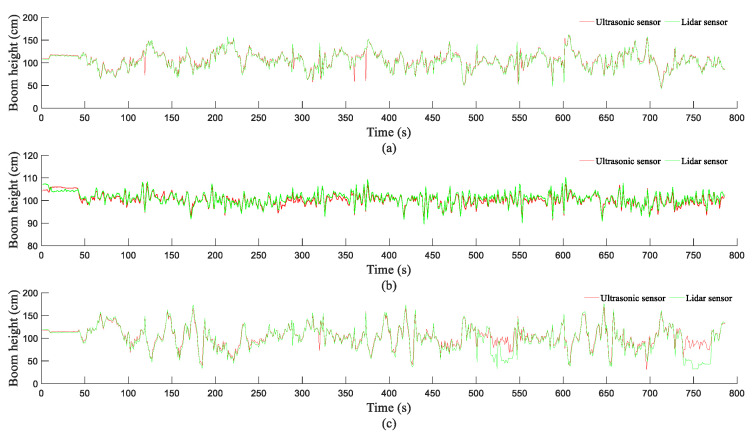

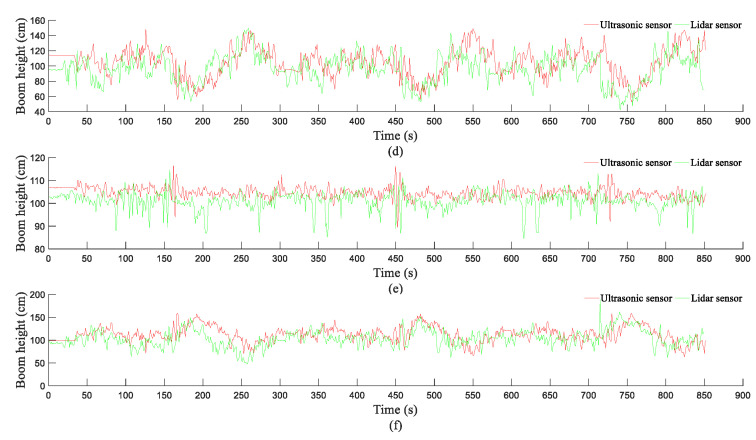
Results of the boom height detection experiment: where (**a**–**c**) corresponds to the *H_b_* changes in the left, middle, and right sections of the boom for rotary-tilled field and (**d**–**f**) corresponds to the *H_b_* changes in the left, middle, and right sections of the boom for the WS field.

Figure 13 shows the *H_b_* values detected by the LiDAR and ultrasonic sensors in the rotary-tilled and WS fields, respectively. As demonstrated in Figure 13, the *H_b_* values detected by the two sensors show similar trends. Currently, ultrasonic sensors are the main type of sensor used to detect *H_b_*, and detection data from ultrasonic sensors can reflect changes in *H_b_* well, which further demonstrates that LiDAR sensors can satisfactorily reflect changes in *H_b_* and can be employed to detect changes in *H_b_*. In addition, the *H_b_* values detected by the two sensors in the rotary-tilled field are basically the same, whereas there is a difference between the *H_b_* values detected by the two sensors in the WS field. Moreover, in the WS field, the *H_b_* values detected by the LiDAR sensor were lower than those detected by the ultrasonic sensor. For a boom sprayer, *H_b_* changes less in the middle section of the boom than at the two ends of the boom. The changes in *H_b_* monitored by the two sensors for the middle section of the boom can reflect the distances detected within the canopy. Figure 13b,e show the *H_b_* values detected by the two sensors in the middle section of the boom. As demonstrated in Figure 13b,e, the distance detected by the LiDAR sensor is lower than that of the ultrasonic sensor within the WS canopy, and the distances detected by the LiDAR sensor for the WS canopy fluctuate to a relatively large extent, which further demonstrates that the distances detected by LiDAR sensor for the WS canopy include the WS canopy and roots.

## 4. Discussion

The comprehensive analysis of the results obtained from the step and field ground detection experiments shows that *V_LiDAR_* affects the detection accuracy of the LiDAR sensor and that there is a delay in the detection signal of the LiDAR sensor when there is a significant step-like change in the distance detected by the LiDAR sensor. However, in the field ground detection experiment, a relatively small detection delay is found in the LiDAR sensor for the slope at which the distance detected by the LiDAR sensor changes continuously. This result suggests that significant changes in the crop canopy height during spraying operations can affect the detection accuracy of the LiDAR sensor. When developing an *H_b_* detection system, we must develop a signal compensation algorithm to address the signal-delay problem found in the LiDAR sensor. Moreover, there is a decrease in the number of sampling points for detection signals when the LiDAR sensor operates at high speeds. Low amounts of sampled data also affect the detection accuracy of the LiDAR sensor. Thus, it is necessary to study high-speed sampling algorithms to increase the number of effective sampling points for the LiDAR sensor. Currently, the ultrasonic sensors are the primary sensors used to detect *H_b_*. There is also a delay in the detection signal of the ultrasonic sensor as the detection speed increases [26]. Meanwhile, there are bottlenecks in the research on signal processing algorithms for ultrasonic sensors for high-speed or complex operating settings. Compared to ultrasonic sensors, the LiDAR sensors have advantages, such as a higher measurement precision, a higher response speed, and a higher interference resistance. The cost of the newly developed LiDAR sensor is approximately $46, which is less than half the price of an existing ultrasonic sensor for boom height detection. The newly developed LiDAR sensor supports research on the boom height detection method and autonomous *H_b_* control systems for high-speed or complex operating settings.

During the wheat harvest, the height of the harvester header can be changed according to terrain changes, and such height changes of the harvester header can lead to the irregularity of the WS canopy. The detection position of the ultrasonic sensors is the surface of the WS canopy [28]. The irregularity of the WS canopy effects the detection accuracy of the ultrasonic sensor. To improve the accuracy of boom height detection, a signal processing method must be added to pre-process the ultrasonic sensor data. For LiDAR sensors, the WS height detection and a comparison of the LiDAR and ultrasonic sensors experiments shows that the LiDAR sensor primarily detected the WS roots and the inside of the WS canopy. *V_LiDAR_* and *H_WS_* has a greater impact on the detection performance of the LiDAR sensor for the WS canopy than that for the WS roots. The detection height values between the boom and WS roots are less affected by the irregularity of the WS canopy. The detection error of the LiDAR sensor for the WS roots is less than 5.00% (the maximum detection error is 4.66%). Thus, in a spraying operation, it is possible to first classify the detection data obtained by the LiDAR sensor for the WS roots and canopy in a field using the K-means clustering algorithm and to subsequently increase the value detected for the WS roots by 5% as a reference basis for adjusting *H_b_* and add the height of the WS canopy to this basis as an index for adjusting *H_b_*. This method can reduce the effects of an uneven WS canopy on the detection accuracy of the LiDAR sensor, which is more suitable for *H_b_* detection in WS field spraying.

In recent years, as boom sprayers are becoming increasingly large and efficient, it is urgently necessary to make breakthroughs in signal processing algorithms for *H_b_* detection sensors or to develop new sensors for *H_b_* detection. Future studies would focus on developing automatic *H_b_* control systems based on the LiDAR sensor designed in this study and conducting field experiments at high speeds and universal experiment of different crop canopies at different stages.

## 5. Conclusions

In this study, a new, low-cost LiDAR sensor for *H_b_* detection was developed based on the principle of single-point ranging. The results obtained from a step height detection experiment, a field ground detection experiment, and a WS height detection experiment as well as a comparison of the LiDAR sensor developed in this study with an ultrasonic sensor showed that the LiDAR sensor could satisfactorily reflect changes in *H_b_* and be used to detect *H_b_*. The LiDAR sensor will promote research on the boom height detection method and autonomous *H_b_* control systems for high-speed or complex operating settings. The distances detected by the LiDAR and ultrasonic sensors differed within the WS canopy, and the LiDAR sensor primarily detected the WS roots and the inside of the WS canopy. The K-means clustering algorithm can be used to classify the heights detected for the WS roots and canopy in a field. In spraying operations, the detection value from the LiDAR sensor to the WS root multiplied by 1.05 can be used as a reference basis for adjusting *H_b_*, and after the WS canopy height is added to the basis, the value can be used as an index for adjusting *H_b_*. This method can reduce the effects of an uneven WS canopy on the detection accuracy of the LiDAR sensor, which is more suitable for *H_b_* detection in WS field spraying.

## Figures and Tables

**Table 1:** Existing boom height detection sensors.

Sensor Type	Detection Index	Sensor Installation Location and Detection Method	Advantages	Disadvantages	References
Ultrasonic sensor	Boom height	The sensor was installed at both ends of the boom to detect the distance between the boom and the ground or canopy	It can directly detect the boom height	Susceptible to external interference	Cui et al. [14,15] Ooms et al. [16,17] Jeon et al. [18]
Tilt sensor	Boom tilt angle	The sensor was installed in the middle of the boom to measure the boom tilt angle	Easy to install	Need to convert the tilt angle into the boom height	Cui et al. [14,15] Qiu et al. [19]
Acceleration sensor	Boom displacement	The sensor was installed at both ends and the middle of the boom to measure the vertical and horizontal vibration displacement of the boom	It can analyse the vibration characteristics of the boom	High installation requirements	Wang et al. [20] Herbst et al. [21]
Touch Sensor	Boom height	The sensor was installed at both ends and the middle of the boom to detect the boom height through contact rod deformation	Fast response and less external interference	The sensor contact rod will damage the crop	Wang et al. [22]
displacement sensor	Boom displacement	The sensor was installed at the connection of the boom and the suspension to detect the displacement of the boom relative to the swing rod and the damper	It can detect the swing displacement of the boom	High installation requirements	Cui et al. [11]

**Table 2:** Parameter indicators of the LiDAR sensor.

Parameter	Value
Detection range	0.1–12 m
Detection accuracy	±1%
Range resolution	1 cm
Signal receiving angle	3.6°
Output frequency	0–1000 Hz (adjustable)

**Table 3:** Mean detection errors of the LiDAR sensor moving at different speeds for the WS roots and canopy in each area.

Distance Detected	*V_LiDAR_* (m/s)	Mean Detection Error for Area a (%)	Mean Detection Error for Area b (%)	Mean Detection Error for Area c (%)
WS roots	0.05	3.24	2.79	1.42
	0.3	2.43	2.45	1.36
	0.6	2.43	3.67	0.68
	0.9	4.66	2.51	1.08
	1.2	2.63	2.51	2.44
WS canopy	0.05	16.80	11.99	6.65
	0.3	18.55	12.84	7.26
	0.6	18.37	11.48	7.65
	0.9	16.80	12.84	8.19
	1.2	20.02	11.31	8.50

## Data Availability

Not applicable.
